# Volar dislocation of second, third, and fourth carpometacarpal joints: a rare and easily missed diagnosis

**DOI:** 10.1007/s10195-012-0181-3

**Published:** 2012-03-06

**Authors:** Javed Jameel, Mohd Zahid, Mazhar Abbas, Abdul Qayyum Khan

**Affiliations:** Department of Orthopaedic Surgery, J.N. Medical College, Aligarh Muslim University, Aligarh, India

**Keywords:** Compound, Volar, Carpometacarpal joint

## Abstract

Volar carpometacarpal dislocation is a rare form of hand injury that can be easily missed without applying a high level of suspicion and performing a meticulous examination. In this case report, we present a rare case of compound volar dislocation of the second, third, and fourth carpometacarpal joints in a 40-year-old male. This was managed by closed reduction and the use of a mini external fixator. The patient regained a good range of motion in 6 weeks with no pain. It is important to diagnose and treat this injury in order to avoid the considerable morbidity associated with this condition.

## Introduction

Volar carpometacarpal dislocations of the fingers of the ulnar side of the hand are a relatively uncommon injury [1, 2], but those involving the middle three metacarpals are rarer still. Diagnosis of this unusual form of injury requires a high level of suspicion, careful examination, and good radiography. Dislocations at the finger carpometacarpal joints are usually high-energy injuries that are commonly seen in boxers and motorcyclists [3, 6]. The diagnosis can easily be missed due to other serious injuries. These injuries account for <1% of all hand injuries [4] and are frequently overlooked or missed. Disability of the hand is severe in untreated cases or in those where treatment has been delayed. Volar dislocations of these joints have been reported on rare occasions, but to our knowledge no such dislocations of just the second, third, and fourth carpometacarpal joints have been placed on record. The case presented here is therefore of significant interest.

## Case report

**Fig. 1:**
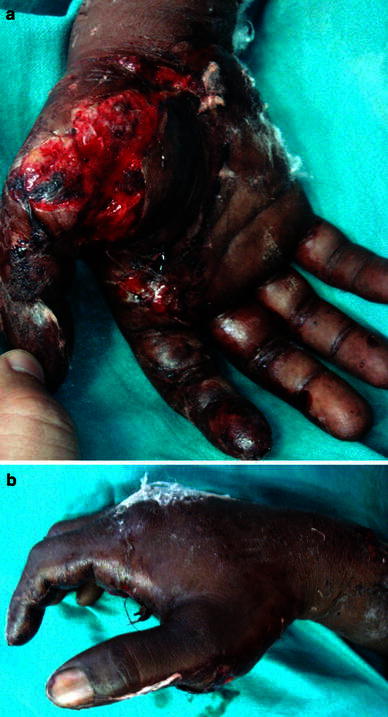
**a** Swelling of the hand with a deep laceration over the thenar eminence. **b** Depression on the dorsum of the hand at the carpometacarpal junction

**Fig. 2:**
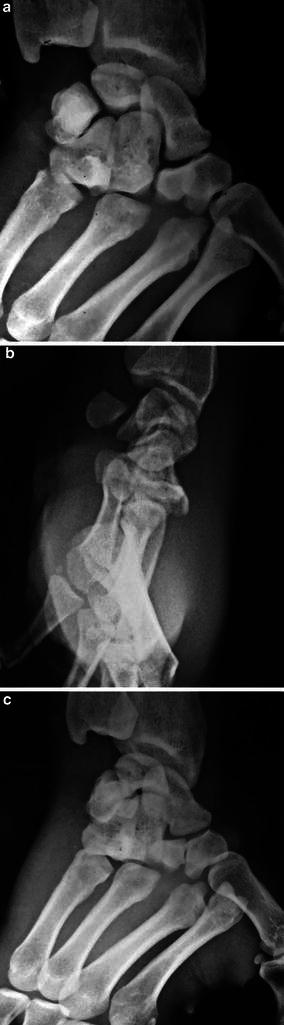
Postinjury radiographs (anteroposterior, lateral, and oblique views) showing volar and radial dislocation of the second, third, and fourth carpometacarpal joints

A 40-year-old man, a shopkeeper by occupation, was admitted to the hospital three days after a high-speed motorbike accident in which he sustained volar-radial dislocation of the bases of the second, third, and fourth metacarpals of the right hand along with multiple abrasions about the forearm. The patient was riding a motorbike that collided with an oncoming truck. He was right-handed and his pre-injury hand function was satisfactory. Examination of the right hand revealed swelling of the hand with a deep laceration (~6 cm) over the thenar eminence (Fig. 1a), diffuse tenderness over the carpometacarpal area, a palpable mass in the palm over the laceration at the ulnar margin of the thenar eminence, and a palpable depression (Fig. 1b) on the dorsum of the hand at the carpometacarpal junction. His distal neurovascular status was intact, but the patient had restricted finger movement because of pain. Roentgenograms of the hand showed volar and radial dislocation of the second, third, and fourth carpometacarpal joints (Fig. 2a, b, and c).

### Surgical procedure

**Fig. 3:**
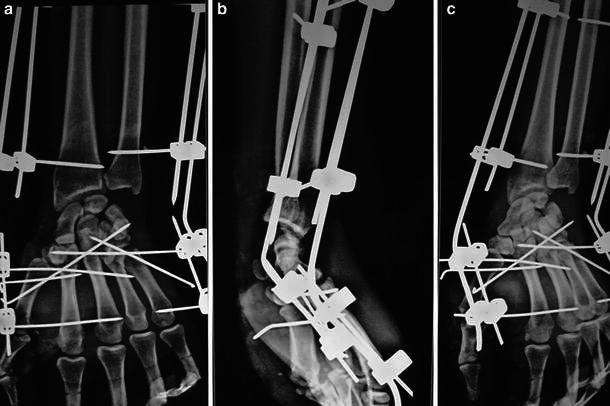
Immediate postoperative radiographs (anteroposterior, lateral, and oblique views) showing the maintained reduction of the second, third, and fourth carpometacarpal joints stabilized with Kirschner wires and the mini external fixator

With the patient supine under general anesthesia, wound debridement was performed, followed by the application of traction to the second, third, and fourth fingers with the elbow being flexed to 90°. The wrist was then acutely flexed while dorsomedially directed pressure was applied to the palm over the bases of the second, third, and fourth metacarpals. Reduction was accomplished by removing the deformity at the carpometacarpal joints, as verified by examining images, and achieved by fixing with multiple 1.5 mm Kirschner wires percutaneously. The wrist was then immobilized by a mini external fixator in a functional position to aid with dressing. Immediate postoperative radiographs (Fig. 3a, b, and c) show the reduced second, third, and fourth carpometacarpal joints stabilized with Kirschner wires and the mini external fixator.

**Fig. 4:**
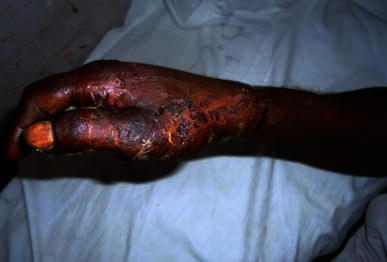
Maintained carpometacarpal relation after the removal of the fixator clinically at 4 weeks post-op

**Fig. 5:**
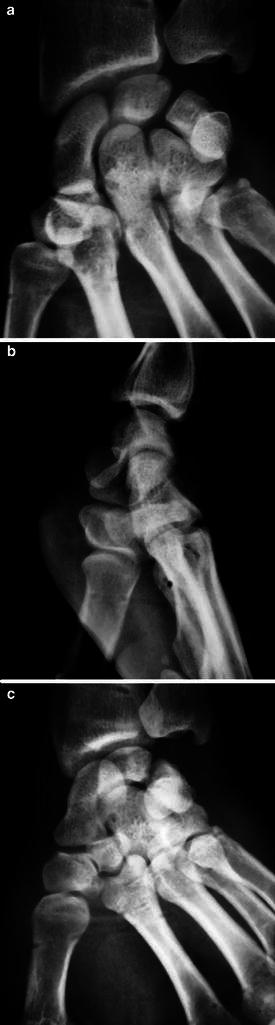
Four-week-postoperative radiographs (anteroposterior, lateral, and oblique views) show maintained carpometacarpal joints of the second, third, and fourth fingers after fixator removal

**Fig. 6:**
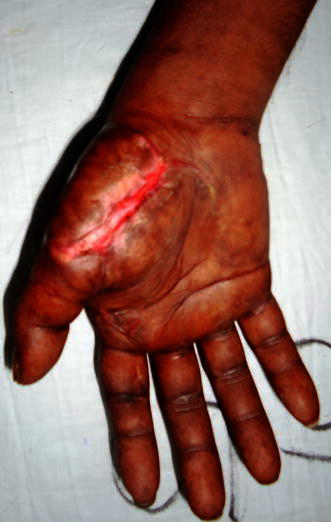
Wound healing at 6 weeks

Daily dressing of the wound and mobilization of the metacarpophalangeal and interphalangeal joints were started the following day. The pins and mini external fixator were removed at 4 weeks post-op, with no reappearance of bony abnormality (Fig. 4). Follow-up X-rays taken at 4 weeks showed the maintained position of the carpometacarpal joints (Fig. 5a, b, and c). The wound healed uneventfully by secondary intention during the next 6 weeks (Fig. 6), and the patient rapidly regained good pain-free ranges of motion of the wrist and fingers and a grip strength that was almost the same as that of the opposite side in the following 6 weeks.

## Discussion

Carpometacarpal dislocations are seen following high-energy trauma. The increased mobility on the ulnar side may predispose to the noted greater frequency of injury. The mechanism of injury in our case would have been a direct thrust over the knuckles that forced the metacarpals to rotate from the dorsal to the volar direction, causing dislocation of the middle three carpometacarpal joints.

The joints between the carpal bones and the second, third, fourth, and fifth metacarpals are all of the modified saddle type. The second and third metacarpals form the rigid central pillar of the hand and are firmly joined to the relatively immobile carpus through their irregularly shaped carpometacarpal articulations. Stability at the finger carpometacarpal joints is provided by a system of four ligaments. They are the dorsal metacarpal, the palmar metacarpal, and the two sets of interosseous ligaments [1]. These injuries are frequently missed initially because of gross swelling of the hand and because overlap on the lateral X-ray obscures the accurate depiction of the injury pattern. Therefore, at least one variant of an oblique view is required for diagnosis in cases with a high level of suspicion [5]. Treatment includes closed reduction, which is usually successful in dislocations <10 days old, and an unstable reduction can be held with percutaneous Kirschner wires. When 3 weeks or more have elapsed since the injury, open reduction will be necessary [6].

To the best of our knowledge, this is the first case of the volar dislocation of the middle three carpometacarpal joints to be reported in the literature. Volar carpometacarpal dislocation is a rare form of hand injury and can easily be missed without applying a high level of suspicion and performing a meticulous examination. All three radiographic views are necessary to make a diagnosis and to avoid the considerable morbidity associated with this condition.
